# The Role of Universities Building an Ecosystem of Climate Change Education

**DOI:** 10.1007/978-3-030-57927-2_1

**Published:** 2020-12-04

**Authors:** Fernando M. Reimers

**Affiliations:** Harvard Graduate School of Education, Harvard University, Cambridge, MA USA; Harvard Graduate School of Education, Harvard University, Cambridge, MA USA

## Abstract

This chapter introduces the field of climate change education, noting the paradox that in spite of many efforts at incorporating climate change in education policy and curriculum frameworks, and a diversity of practices in schools, there is little evidence that such efforts are contributing to adaptation, mitigation or reversal of climate change. The chapter reviews the role of international development organizations advocating for and developing frameworks in support of climate change education. This is followed by an analysis of ongoing efforts of climate change education.

The chapter argues that more effective education for climate change at the primary and secondary education levels around the world requires context specific strategies that align the specific learning outcomes with the impacts of climate change in that context. Implementing those strategies requires the development of institutional capacity in schools that is aligned to the stage of institutional development of the school. The chapter explains how a multidisciplinary framework that accounts for the cultural, psychological, professional, institutional and political dimensions of the change process can support the development of collaboration and coherence in implementing those climate change education strategies. Those strategies need to also specify the particular populations that need to develop such competencies and the optimal means of delivery. The chapter also situates the literature on climate change education within the larger context of the literature on deeper learning, twenty first century skills and education system change, explaining how deeper learning in climate change education might influence attitudes and behaviors in ways that prevailing didactic approaches focused principally on the transmission of scientific knowledge do not.

To develop such context specific climate change education strategies and to build the institutional capacity to implement them, the chapter makes the case for more intentional engagement of universities, in partnership with schools and non-formal education organizations. This would serve the dual role of providing support for schools in advancing climate change education, while also educating higher education students on climate change through problem based, participatory and contextually situated approaches.

## Introduction. The Paradox of Climate Change and Education

Along with many species on the planet, polar bears are experiencing the effects of climate change. As rising temperatures produce ice loss, the bears lose the platforms they use to hunt seals. This change to their habitat has placed polar bears on the list of species on the risk of extinction in the wild (Pidcock 2015). The bear floating on a melting ice platform has become an icon for the potentially devastating consequences of climate change to life on the planet.

Like bears, humans also face risks to their habitat and survival resulting from climate change, such as the intensification of the wildfire season ravaging the West Coast in the United States, or more intense storms, droughts and floods, rising sea levels or increasing temperatures. Unlike bears, however, humans have invented an institution to help us quickly adapt to and mitigate changing trends: schools. Furthermore, because climate change is largely the result of human-environmental interactions (IPCC 2018, p 53), schools can do more than help us understand these changes to our habitat, or help us adapt to those changes, they could help us slow down those changes and mitigate their impact, as we adopt practices that are more sustainable, and perhaps even revert them, as we invent technologies that transform the drivers of climate change.

The International Panel on Climate Change (IPCC), has established that human activities have caused global temperature to rise about 1.0 °C degrees above preindustrial levels (before the 1880s) and that, if increases continue on the current trajectory, global warming will likely reach 1.5 °C between 2030 and 2052. These changes to climate pose risks to health, livelihoods, food security, water supply, human security, and economic growth, and will increase as temperatures reach 1.5 °C and increase further as temperature increase to 2 °C above preindustrial levels. The impact of global warming of 1.5 °C and beyond will be greater on disadvantaged and vulnerable populations, indigenous peoples, and communities whose livelihood is dependent on agricultural or coastal activities. Global warming will also contribute to increases in poverty (IPCC 2018, p. 4–9.) and will have a disproportionate impact on women who are poor and from other disadvantaged groups and whose livelihood depends largely on agriculture.

“Differences in vulnerability and exposure arise from non-climatic factors and from multidimensional inequalities often produced by uneven development processes (very high confidence). These differences shape differential risks from climate change. People who are socially, economically, culturally, politically, institutionally or otherwise marginalized are especially vulnerable to climate change and also to some adaptation and mitigation responses (medium evidence, high agreement). This heightened vulnerability is rarely due to a single cause. Rather, it is the product of intersecting social processes that result in inequalities in socio-economic status and income, as well as in exposure. Such social processes include, for example, discrimination on the basis of gender, class, ethnicity, age and (dis)ability.” (IPCC 2014, p 54).

The IPCC identifies a range of education options to adapt to and mitigate climate change, including awareness raising and integration of climate change education in school curricula, gender equity in education; and various forms of adult and non-formal education, including extension services; sharing indigenous, traditional & local knowledge; participatory action research & social learning; Knowledge-sharing & learning platforms and disseminating information on hazards and vulnerability (IPCC 2014, p.27).

Educating people for more sustainable ways to relate to our habitat involves preparing us to adopt sustainable practices that reduce our impact on climate change and the impact of climate change in our lives. These practices may be individual, in the choices we make about our own consumption and lifestyle (for example slowing down population growth, consuming a diet with a smaller carbon footprint or using renewable energies, or consuming less), or they may be collective, the result of choices we make as citizens when we participate in the democratic process at various levels of government, our towns or cities, states, or nations, or when we influence the behavior of corporations (for example adopting caps to emissions or a carbon tax, or incentivizing the reliance on clean energies). Government policies such as caps on emissions are essential to slowing global warming, and they are subject to influence and preferences by citizens, educated to understand the scientific consensus on climate change and with the capacity to exercise influence as citizens. Collective responses may also include shaping the way in which we live and our habitats, for instance the value we assign to nature as we design and build the homes and cities where we live and work.

In addition to personal responsibility for our individual impact on climate change, and participation in collective processes that support systemic changes in the norms and institutions that undergird climate change, slowing down, and perhaps over time reverting, climate change requires also advancing knowledge and inventing technologies that can help us transform our interactions with the environment, in a way helping us reinvent our way of life, and so educating for sustainability involves equipping people with the ethical frameworks, the imagination and the necessary skills for such advancement of knowledge and invention. Example of such design and invention and changes to our way of life include developing a circular economy with production of goods next to cities to reduce transportation costs, as well as urbanization with populations concentrated in sustainable cities, or geoengineering the atmosphere to partially block the sun’s rays.

An example from the field of sanitation will illustrate the value of technological invention to address climate change. In his efforts to improve health and sanitation in the developing world, Bill Gates concluded that the toilets and water treatment systems developed and in use in the early industrialized world were inadequate to improving sanitation in developing countries because they were resource intensive, generated excessive waste and required intricate and expensive sewer systems to operate. As a result, as an approach to dispose of human waste, toilets are likely to remain out of the reach of a significant share of the world’s population. This caused him to undertake projects to stimulate innovation in the design of next-generation toilets that could operate without sewer systems and that could be extended to all of humanity within a relatively short period of time (Brueck 2019; D’Agostino 2018). Similar technological breakthroughs could change our dependence on fossil fuels, help us produce much safer nuclear energy, increase the efficiency of fossil fuels and of clean energies. But it is not just technological advancements that can help us reinvent a way of life, inventions in how we organize our lives and work, and in how we organize our communities can help us mitigate and adapt to climate change. For example, structuring some workplaces in ways that allows working from home can reduce our consumption of fuels. Ethical and spiritual development can stimulate such social innovation and lead us to make different choices placing different value on individual consumption relative to protection of the environment, other forms of life or cause us to seek greater balance across a range of goals in the communities of which we are a part. The United Nations Sustainable Development Goals, for example, are a framework of seventeen interdependent goals that aim at producing a world that is more inclusive and sustainable, and they can provide a normative framework to guide the development of communities, cities, or other jurisdictions.

The motivation to invent more sustainable ways of life requires more than an understanding of the science of climate change, the capacity to design technological innovations, or an ethical framework that help us aspire to live in more inclusive and sustainable communities, it requires an understanding of social systems and the development of ethical reasoning that can help us integrate critical thinking about the current impact of climate change, our moral imagination, the personal motivation to act and our competency to act in effective ways. An example of the integration of understanding of complex social systems with ethical reasoning would be engaging students in projects that helped them understand the gendered experience of climate change. A number of reports explain that the dependence of women in developing countries on natural resources makes them particularly vulnerable to climate change. The challenges are greater for women who secure water, food and fuel for cooking and heating and whose livelihood depends on agriculture. A number of studies of the gendered nature of climate change argue that these differences are the result of various intersectionalities that place particular groups of women (poor, lower casts) at greater risk (Arora-Jonsson 2011). Therefore, understanding intersectionality is necessary to better understand the gendered impact of climate change. The drivers of these gender differences include disparities in access to education, use of time, access to credit and markets, recognition of rights within legal frameworks, and resulting disparities in earnings (UNDP 2013) so understanding these drivers requires understanding systems and complex causality. The recognition of the gendered impact of climate change is the foundation for the recognition of the co-benefits between gender equality and climate action (UN Women 2016) this provides an opportunity for students to understand deeply complex social action and how “equity, sustainable development, and poverty eradication are best understood as mutually supportive and co-achievable within the context of climate action and are underpinned by various other international hard and soft law instruments” (IPCC 2018, p 54).

Developing the moral imagination of students through Human Rights education or education for social justice, cultivating their capacity to recognize how the impact of climate change varies for different people (women, minorities, the poor) is a necessary step to animating them to engage with the subject at greater levels of complexity and inventiveness.

As illustrated with the previous discussion of the complementarities between gender equity and climate change, given the multidimensional nature of the impacts of climate change underscored in recent reports of the IPCC, effective collective responses require addressing the systems that undergird such multidimensional processes. This understanding has led to a growing realization that climate action is best undertaken in coordination in the context of poverty reduction and sustainability efforts, such as those reflected in the development compact adopted at the UN 2015 General Assembly: the Sustainable Development Goals. Advancing such systemic multidimensional efforts requires that we educate students to understand systemic complexity, and develop their capacity to collaborate with others to influence social systems.

“Differences in vulnerability and exposure arise from non-climatic factors and from multidimensional inequalities often produced by uneven development processes (very high confidence). These differences shape differential risks from climate change. People who are socially, economically, culturally, politically, institutionally or otherwise marginalized are especially vulnerable to climate change and also to some adaptation and mitigation responses (medium evidence, high agreement). This heightened vulnerability is rarely due to a single cause. Rather, it is the product of intersecting social processes that result in inequalities in socio-economic status and income, as well as in exposure. Such social processes include, for example, discrimination on the basis of gender, class, ethnicity, age and (dis)ability.” (IPCC 2014, p 54).

To sum up, human competencies, the knowledge, motivation and skills of people, are critical to adapting to and mitigating climate change. Developing those competencies is, however, a very tall order, one that requires focus and specialization.

This potential of education to affect human-environmental interactions has given rise to a new educational domain of education: climate change education, a subfield of education for sustainable development. Much has been written, and is being done, to educate students to understand, adapt to and mitigate climate change. Governments around the world, in partnership with organizations of civil society and with other institutions, have developed climate change curriculum and adopted policies to address this serious risk faced by humanity. International organizations, UNESCO in particular, have advocated extensively for climate change education and developed and distributed resources to support it. In spite of these efforts, education has not yet sufficiently curbed the impact of our own species on climate change, nor have we yet adapted to these climatic changes and as a result, like polar bears, we are witnessing the destruction of our habitat, much of such destruction of our own doing, and wondering whether we will, along with other species, survive such changes.

“Short of some technological revolution that would transform global energy use, we should be concerned, even alarmed, about the future impact of climate change on the world. It is the quintessential global challenge in that no single country can solve this problem on its own and there is no way for any single country to shield itself from its effects. Generating the required collective response, however, seems highly unlikely. As a result, climate change could conceivably be the defining issue of this century.” (Haas 2020, p. 192).

## Climate Is Changing Faster Than Attitudes and Behaviors About Human-Environmental Interactions, and Knowledge Is Not Enough to Cause People to Adapt or Mitigate

While there is arguably more interest around the world in environmental sustainability, and in climate change, than at any time in human history (Mayherfeld and Askhood 2015), it is also the case that our climate challenges are greater than ever. The question then is not whether the environmental movement has increased awareness and action to address climate change, the question is whether it has done so on a scale and level of effectiveness commensurate with the nature of our present challenge and with the velocity at which the challenge is augmenting.

A recent survey of sustainable development experts and practitioners from business, government, NGOs and academia reveals that more than half of those surveyed believe that the rate of progress with respect to climate change is insufficient to avert major damage to human, social and ecosystem health, and less than a third of them believe that good progress is being made implementing the global framework adopted in the Paris agreement (GlobeScan 2017, p. 4). Furthermore, climate optimism (the belief that society is making progress fast enough to avert major irreversible damage to human, social and ecosystem health) has declined considerably over the last fifteen years. In North America, close to 20% of respondents believed it likely or very likely that we were making adequate progress in 2003, compared with 11% in 2017. Climate optimism, has also declined for the rest of the world, from 11% in 2003 to 5% in 2017 (GlobeScan 2017, p. 11).

The most recent World Economic Forum Report on Global Risks identifies climate related risks as the most likely of all risks humanity faces. They include extreme weather, climate action failure, natural hazards, biodiversity loss and human made environmental disasters. Of those, climate action failure, biodiversity loss and extreme weather are also among the five most potentially impactful risks (World Economic Forum 2020).

Climate change, observable changes in climate patterns resulting from a warming of the temperature of the atmosphere, is the result of human activity, principally burning of fossil fuels which release carbon dioxide and other greenhouse gases into the atmosphere where they trap the sun’s rays, thereby increasing temperature. The five warmest years since 1880 have all occurred since 2015 (NOAA 2020). Climate change is causing increases in sea levels in coastal areas, more severe storms, higher temperatures and increasing desertification and wildfires. This will reduce productive land. Rising temperatures and salinization will endanger many life forms, crop yields and disease prevalence. Freshwater shortages, extreme heat, flooding and storms will cause large scale migration. The two main drivers of climate change are consumption of fossil fuels and deforestation (Haas 2020, pp. 183–186).

Increases in consumption of fossil fuels stem from the growing levels of consumption and waste produced by a growing population and by the energy and waste produced by modern manufacturing. Consumption, population growth, energy and waste are therefore the major drivers of climate change producing significant release of carbon dioxide and other bases into the atmosphere which trap heat. The resulting warming of the planet is causing rapid and extensive biodiversity loss and land degradation (UNEP 2012; UNESCO 2016). Over the last fifty years fossil fuel consumption has tripled, largely a result of increases in transportation, construction and industrial manufacturing (Haas 2020, p. 185).

Scientists have identified boundaries for ten systems that affect life for humans and other species: freshwater use, land use, phosphorus pollution, ocean acidification, climate change, ozone depletion, nitrogen pollution, biodiversity loss, aerosols and chemical pollution. While we have no data on how aerosols and chemical pollution has changed since preindustrial levels, for eight of those system metrics for which we do have data to compare pre-industrial revolution levels to current levels, five of them exceed the boundaries representing high risk that life is not sustainable. These systems are: ocean acidification, climate change, ozone depletion, nitrogen pollution, biodiversity loss. Furthermore, the remaining three metrics: freshwater use, land use and phosphorus pollution, have changed significantly, in the direction of the increasing risk boundary. Only two of the eight metrics (ocean acidification and ozone depletion) have current values which are lower than the values before the industrial revolution, although they remain above the proposed boundary representing high risk (UNESCO 2016, p. 20). The most commonly accepted explanations for those changes focus on overpopulation, modern lifestyles and individual behavior (UNESCO 2016), as well as industrialization and the release of carbon dioxide in the atmosphere.

Population growth, and the energy and resources that more people consume, is a major driver of climate change. It took one thousand years for the world population to grow from an estimated 190,000,000 in the year 200 to 360,000,000 in the year 1200, and another six hundred years to reach one billion in the year 1804. But the improvement in life expectancy associated with medical and public health developments grew the world population by an additional billion people in barely a century. Then, in less than four decades, world population grew from 2 billion in 1927 to over 3 billion in 1960. The next additional billion in world population took only fourteen years, exceeding four billion in 1974. The next billion humans took only thirteen years, the next billion twelve years, and the next additional billion another twelve years, until the world population reached more than seven billion by 2011 (Worldometers 2019). Such exponential growth in the number of humans making demands on the planet is a driver for the changes to the environmental systems discussed earlier. Some of those demands on natural resources and on the atmosphere are the product not just of the number of people but of particular forms of consumption, of forms of life and social and economic organization. For instance, construction, transportation and manufacturing account for most of our consumption of fossil fuels. Circular economies and alternative forms of urbanization can reduce these costs of transportation and construction.

Providing individuals with access to jobs and income, so they can sustain those forms of consumption, and the necessity to grow aggregate economic output to expand such access in the face of a growing population, has been a widely accepted view of ‘progress’ for many nations for much of the twentieth century. Only during the last decades of the century did the notion that there may be tradeoffs between jobs and consumption, or economic growth and sustainability, become increasingly accepted among government leaders and development professionals, this shift was significantly aided by the United Nations Conference on Environment and Development (Earth Summit) which took place in June 1992 in Rio de Janeiro, Brazil (UNCED 1992). The question of what constitutes development, and how to integrate sustainable development with other social and economic goals, such as poverty reduction and improvement in material standards of living, is at the heart of today’s conversation about what is sustainable development. As with climate change, if such conversation is to have any consequence on the ten systems which are moving the planet towards levels of increased risk for life, it needs to move beyond a conversation among elites in governments and international organizations, and become part of how most humans on the planet think about what is a good life and how they make individual and collective choices that lead to greater sustainability. For instance, governments could agree on a total amount of emissions or on a tax of emissions, something no government has yet endorsed (Haas 2020, p. 189). Similarly, individuals could choose to protect natural habitats and consume less, including reducing their living space, or change their diet, as a way to reduce their carbon footprint.

Climate change education must equip most humans with the knowledge, critical thinking skills, understanding of science, and ethical frameworks, that helps them with mitigation, adaptation and reversal of climate change. Mitigation involves trying to slow down the rate of climate change. Adaptation involves reducing the impact of climate change on people. Reversal is a nascent area involving geoengineering, such as planning particles in the atmosphere to partially block sun’s rays (Haas 2020, p. 191).

This means that climate change education should go beyond equipping people with the skills to understand climate change, important as that is. It must equip them to understand tradeoffs, to make choices and to invent solutions that can help us integrate choices that are environmentally sustainable within a larger framework of how we live. A simple way to represent the choices involved in mitigation would be to ask how much people are willing to give up, or to pay, or to change their lifestyle, to reduce their own carbon footprint, for example demanding that their governments adopt cap and trade that limit the total emissions or a carbon tax. Educating people for adaptation involves knowledge and skills to change their lifestyles in ways that are responsive to the impact of climate, for example influencing how they build or rebuild their cities so they are more resilient to coastal flooding. Finally, educating them for climate change reversal requires developing the talent and ingenuity that can lead to technological innovations. This means education not just to understand, but to act effectively, in influencing not just personal patterns of consumption, but with the agency and efficacy to collaborate with others to influence the complex systems which undergird climate change.

Awareness and knowledge about climate change appear to be insufficient because while there is evidence that a significant percentage of people are already aware of environmental degradation, and consider it a challenge, climate continues to change. The World Values Survey, a project coordinated at the University of Michigan, collects data from representative samples of the population in a large number of countries on a range of issues. When people were asked to identify the most important world problem, environmental degradation is considered one of the most important problems in the world, second only to poverty which is considered the most important problem by most people in most countries. Environmental pollution is considered the most important world problem in more countries than discrimination against women, and also more important than poor sanitation and infectious diseases. Only with respect to poor education is the number of countries that see this as the most important problem in the world similar to those who see environmental degradation as the most important problem. The results can be seen in Table 1.1.

**Table 1.1:** Percentage of representative samples of adults who see the following as the most serious problem in the world 2010–2014

	People living in poverty and need	Discrimination against girls and women	Poor sanitation and infectious diseases	Inadequate education	Environmental pollution
Total	56.5	7.8	10.2	11.6	12.7
Algeria	50.7	9.6	16.6	8.8	10.9
Azerbaijan	52.8	11.7	11.3	15.6	7.8
Argentina	59.3	8.3	3.6	18.8	9.1
Australia	61.6	4.2	10.3	12.1	11.5
Armenia	74.7	2.1	7.1	3.1	10.6
Brazil	59.2	10.1	10.5	14	5.8
Belarus	61.5	3.3	10.3	3.1	21.3
Chile	61.2	5.9	5.5	21	5.8
China	42.5	5.1	8.5	12.1	21.6
Taiwan	39.9	3.5	10.4	6.1	36.2
Colombia	56.8	14	2.1	13.6	13.5
Cyprus	57.3	6.3	18.7	5.8	11.9
Ecuador	43	8.8	6.9	18	23.1
Estonia	60.3	2.8	10	5.9	20.2
Georgia	73.1	5.2	8.2	4.7	7.7
Palestine	68.9	5.4	6.7	8.4	9.7
Germany	55.8	7.6	6.5	19.3	10.3
Ghana	58.5	6	14.2	16.8	4.5
Haiti	55.5	15.1	7.8	18.8	1.7
Hong Kong	35.3	5	21.6	12.1	25.6
India	58.5	15	4	14.7	7.5
Iraq	60.8	5.8	7.5	12.8	12.2
Japan	36.6	0.7	7.2	8.1	41.3
Kazakhstan	62.6	3.4	11.8	8	14.2
Jordan	80.7	2.5	5.2	7	4.2
South Korea	42.3	6.3	7.4	4	39.5
Kuwait	49.1	5.4	13.3	21.7	7.3
Kyrgyzstan	54	11.6	13.4	8.7	12.3
Lebanon	48	7.2	17	15.2	11.5
Libya	29.2	3.7	28	26.5	11
Malaysia	44.1	10.9	12.4	16.2	16.5
Mexico	44.4	14.1	5	15.4	20.3
Morocco	62	5.6	6.5	17.6	6.9
Netherlands	64.2	11.6	7.8	5.6	10.3
New Zealand	53.4	3.2	9	15.5	14
Nigeria	79.9	8.2	5.3	5.4	1.2
Pakistan	62.6	20.5	7.8	8.6	0.4
Peru	48	7.4	3.8	22.1	18
Philippines	54.8	5.5	9.8	13.5	16.3
Poland	76.5	2.7	4.3	4.2	11.1
Qatar	52.8	2.6	17.2	8.3	18.8
Romania	52	7	13.7	17.3	8.7
Russia	55.7	3.7	9.9	5.3	22.7
Rwanda	60.4	14.2	19.3	3.3	2.8
Singapore	53.5	11.1	15.3	7.9	12.1
Slovenia	68.4	3.3	4.3	4.3	17.9
South Africa	57.4	17.3	12.7	8.8	3.8
Zimbabwe	57.4	8.1	20.6	11.1	2.8
Spain	71.6	8.4	6.1	8.7	5.2
Sweden	55.1	9	7.9	6.7	20.3
Thailand	46.8	11.8	11.9	12.4	16.9
Trinidad and Tobago	59.2	7.7	10.1	11.5	10.9
Tunisia	84.6	1.9	3.2	5.6	4.1
Turkey	51.6	12	7.6	22.8	4.1
Ukraine	63.4	3.3	12.1	2.3	19
Egypt	81.5	2.2	5.9	8.1	2.2
United States	53.1	4	13	18.9	9.9
Uruguay	56.9	12.7	5.2	14.7	9.4
Uzbekistan	40.6	4.4	25	5.7	21.8
Yemen	74.3	1.8	5.7	13.4	3.6

Source: Inglehart et al. (2014). World Values Survey Database

However, even though people are aware of the importance of environmental sustainability, there is less evidence that they have the skills to translate such awareness into actions that contribute to more sustainable ways of living. This is because human behavior with respect to relating to the environment is not just a function of what we know about that relationship, but about how we weigh the tradeoffs involved in relating in different ways. People may be aware of the fact that walking or riding a bicycle have a smaller carbon footprint than using other forms of transport, and still prefer the convenience, or the income they could draw from the time saved, associated with public transportation or even private transportation. Few workplaces allow extensive telecommuting for work even though doing so would reduce the carbon impact of transportation, and of office space. There are today, effective alternatives to air travel, in the form of highly effective telecommunication technologies with a lower carbon footprint, and yet many people and organizations continue to depend on in person meetings that require air travel, including meetings to discuss how to address climate change.

It is the way most people respond to those tradeoffs that is of greatest consequence to how we relate to the environment, not just the simple ignorance of the facts about the consequences of our actions or knowledge of our alternatives. Existing evidence suggests we are still very far from living in a world in which most people are prepared to value environmental sustainability over other desired goals, such as high levels of consumption, or jobs. One of the questions in the World Values Survey asks respondents to choose between the statement that ‘Protecting the environment should be given priority, even if it causes some loss of jobs’ and ‘Economic growth and creating jobs should be the top priority, even if the environment suffers to some extent’. For all the countries participating in the survey, on average only 47% of the respondents favored the environment over jobs. The percentage who responded this way varied across countries as seen in Table 1.2, from a high of 74% in Malaysia, to a low of 4% in Haiti.

**Table 1.2:** Percentage of the population who thinks that protecting the environment should be given priority even if it causes some loss of jobs between 2010 and 2014

Country	Percentage
Malaysia	73.6
Colombia	67
Chile	66.7
Philippines	64.9
Uruguay	64.2
Qatar	63.1
Sweden	62.9
Peru	62.9
Mexico	62.8
Uzbekistan	62.1
Ecuador	61.2
Taiwan	60.6
Brazil	60.3
Kyrgyzstan	59.3
Georgia	59.1
Australia	59
Hong Kong	58.6
India	58.4
Thailand	57.5
China	56.6
Belarus	56.2
Libya	54.5
Argentina	54.2
Kazakhstan	53.9
Morocco	53.2
Ghana	50.3
Russia	50.2
South Korea	48.2
Turkey	48
Ukraine	47.7
Germany	47.7
Estonia	47.7
Cyprus	47
Palestine	46.8
Pakistan	45.7
Slovenia	44.5
Iraq	43
New Zealand	42.6
Singapore	41.1
Netherlands	40.9
Armenia	40.1
Lebanon	39.9
South Africa	38.3
Poland	37.6
Zimbabwe	37.3
United States	37.2
Jordan	35.8
Spain	35.2
Romania	34.8
Nigeria	33.8
Yemen	33
Tunisia	32
Algeria	31.2
Azerbaijan	31.1
Egypt	30.5
Kuwait	27.2
Japan	22.7
Rwanda	22.1
Haiti	3.8

Source: World Values Survey database. Inglehart et al. (2014)

The relative value people assign to environmental protection over jobs and growth is related to the economic opportunities available, as demonstrated by the case of the United States. Each year, since 1985, the Gallup organization has asked representative samples of Americans whether the environment should be protected, when this goal conflicts with economic growth. During the thirty-four year period over which these data have been collected, a greater priority was given to economic growth during the years when unemployment rates were higher. The figures reported in the previous table from the World Values Survey for the United Sates correspond to a peak in unemployment, over 8% between 2010 and 2013. In 2019, a period of low unemployment at 4%, most respondents, about three in five, agree that environmental protection should be prioritized over economic growth. These fluctuations notwithstanding, however, the percentage of the population who thinks the environment should be protected is very similar in 2019 (65%) to levels in 1985 (61%), even though education and public awareness of climate change have increased during this period (Saad 2019). It is also unclear whether the level of education greatly impacts those choices, though it presumably impacts knowledge of climate change. In the United States, college graduates are only slightly more likely to favor protecting the environment (67%) than those without college degrees (62%).

It is possible that the reason there are few differences in how people with different levels of education, and presumably knowledge, value the environment, is because values, rather than knowledge, play an important role. There are significant differences by age and political affiliation in how likely people are to favor the environment, which suggests that these are valued based choices, not just knowledge-based choices. Among those aged 18–24, 78% would favor environmental protection when it conflicts with economic growth, compared to 58% among those aged 25–54 or to 60% for those over 55. Among Republicans, 35% would favor environmental protection over economic growth, compared to 71% among independents and 82% among democrats. This suggests that if education is to influence how we relate to the environment, it must activate our moral imagination, our capacity for ethical and critical thinking and not just dispense us with more facts.

In addition to the limited predictive value of knowledge and awareness over individual behavior, addressing climate change effectively requires more than influencing the private choices of individuals. Climate change is the result of systems, of production and consumption, and influencing systems requires not just understanding them, but the skills to work with others in inducing change in those systems, not just changes on individual behavior.

## Climate Change Education

A great variety of approaches and methods co-exist under the same term of ‘Climate Change Education’. A surface understanding of the term involves teaching students to understand an existing, scientifically established phenomenon, the result of the way in which humans relate to the environment. Indeed, many of the practices in the field represent didactic approaches of teaching facts of that sort. A rich example of that didactic approach is a complete Climate Change Curriculum developed as a collaboration between climate scientists and teacher education faculty at Stanford University, and middle and high school teachers in the San Francisco Bay Area. The curriculum teaches climate science, the impacts of climate change on society and on global resources and mitigation and adaptation strategies (Stanford University 2020).

This approach to climate science education, as teaching the science of climate change, is rooted in the origins of environmental education, a field which emerged in the 1960s. The seminal contributions of Albert Baez, the physicist who initiated UNESCO’s efforts to promote science education in the developing world, are illustrative of the interest of scientists on environmental issues at the time. Baez proposed in the 1960s that science education had to help students advance the purposes of Peace, Poverty Reduction, Pollution Reduction and Over Population (Reimers 2007).

This approach of climate science education, and environmental education more generally, as anchored in science education dominates the field to date, as shown by a review of 220 studies of climate change education conducted between 1993 and 2014 which identified that most of them approached climate change as STEM education (science, technology, engineering and mathematics), or with environmental and sustainability education (Rousell and Cutter-Mackenzie-Knowles 2020, p. 198).

This early work to include environmental themes within scientific literacy was stimulated by and reinforced by attention to climate and sustainability by other UN agencies. In 1972, the United Nations convened the first Conference on the Human Environment in Stockholm, which would become a milestone in the global environmental movement. A review of the history of research in environmental education concludes that the UN efforts on environmental issues supported the interest in environmental education (Gough 2013). The same review argues that, over time, UN agencies shifted the field from environmental education, to education for sustainable development, with more emphasis on cultivating human capacities to address environmental and development challenges.

In 2010, for example, UNESCO launched the Climate Change Education for Sustainable Development program as an effort to foster ‘climate literacy’ among students (UNESCO 2010). The initiative called for four integrated programs: (1) Climate science and knowledge, (2) Climate change education, (3) Climate Change, Cultural and Biological Diversity, and Cultural Heritage, and (4) Climate change, ethics, social and human sciences dimensions.

As the field of climate science education, and education for sustainability, evolved, so did interest on a broader range of outcomes beyond knowledge and on student centered, participatory, cross disciplinary and multidimensional approaches. Illustrative of those are ‘whole school’ approaches, these are the product of the ‘green school’ movement, which traces its roots to the 1992 UN Conference on the Environment and Development. These green schools or eco-schools are integrated in networks which form a movement aimed at influencing sustainability in the societies where they operate (Gough et al. 2020, in press). The latter is the approach advocated by UNESCO in a guide to support climate change education:

“In a whole-school approach, students’ classroom learning about climate change is reinforced by the formal and informal messages promoted by the school’s values and actions. In other words, students – girls and boys alike - and other members of the school community live what they learn, and learn what they live. The whole-school approach to climate change means that an educational institution includes action for reducing climate change in every aspect of school life. This includes school governance, teaching content and methodology, campus and facilities management as well as cooperation with partners and the broader communities.” (Gibb 2016, p. 3).

UNESCO’s guide for whole school climate change education emphasizes a six-steps process, and is relatively thin on curriculum and content. The process comprises creating a school climate action team, infusing sustainable development across all subjects, teaching creative and futures thinking, empowering students to take action, address facilities and operations and build community partnerships. In terms of curriculum, the guide offers two examples of activities which could be integrated into each of eleven different subjects, such as designing and maintaining a school garden and compost, creating maps showing areas of the world most at risk due to climate change and examining how societies throughout history have resolved conflicts and responded to environmental challenges (Gibb, 2016 p. 12).

UNESCOs Climate change education program proposed using

“innovative educational approaches to help a broad audience (with particular focus on youth), understand, address, mitigate, and adapt to the impacts of climate change, encourage the changes in attitudes and behaviours needed to put our world on a more sustainable development path, and build a new generation of climate change-aware citizens.” (UNESCO 2010, p. 4).

As part of their advocacy for Climate Change Education, and Education for Environmental Sustainability more broadly, the UN and its specialized agencies routinely ask governments to report on the extent to which these topics are included in policies and in the curriculum of instruction in countries around the world. The reports provided by governments to international development agencies suggest that these themes are increasingly recognized by policy and included in the curriculum, which provides an authorizing environment to support instructional practice, but does not necessarily produce high quality or effective practice as will be discussed later in this chapter.

For example, a recent survey administered by UNESCO to member states assessing the extent to which the national curricula in 82 nations (representing about half of the countries surveyed) addressed environmental topics revealed that most governments report that topics such as Climate change, Environmental sustainability, caring for the planet, sustainable development, consumption, and livelihood are included in the national curricula (UNESCO 2018, Figure 6).

In China, Education for Sustainable Development was incorporated in the national standards in 2010. Denmark included climate change education and education for sustainable development in the curriculum in 2009. Recent legislation in Italy mandates the introduction of a required course on climate change (Berger 2019).

“In India, for example, environmental education was mandated by the Supreme Court in 1991, and in 2003 the government directed the National Council of Educational Research and Training to produce extensive content on environmental education.” (UNESCO 2016, p. 25).

In the United States, the new science standards, a set of standards for voluntary adoption by States developed by the National Research Council, the National Science Foundation, the American Association for the Advancement of Science and the National Science Teacher Association, have introduced the subject of climate change in elementary school, with opportunities for deeper study in middle and high school (Chen 2017).

While it is significant that the environmental education movement has gained traction on government policy statements, governments’ self-reports to UN agencies following up on inter-governmental agreements have inherent limitations. In addition, governmental commitments to climate change education are fluid, the subject of the partisan contestation that surrounds the topic of climate change itself.

Illustrative of the volatility in the policy priority afforded climate change education is the case of Australia. Over the last two decades, Australia has advanced a number of initiatives on education for sustainability, adopting a national plan of environmental education in the year 2000, focusing more intentionally on environmental sustainability in 2006, and in 2008 increasing the focus on climate change in the Melbourne Declaration: a statement on educational goal for young Australians subscribed by all education ministers (Ministerial Council on Education 2008). The topic, however, remains contentious and many of these efforts were discontinued in 2013. A 2019 report on the state of climate change education in Australia states that the country lacks a coherent strategy for climate change education and that teachers are left to fend for themselves in addressing the subject (Whitehouse 2019). A recent analysis of the state of climate change education in Australia, notes that the references to climate change and integrating sustainability across the curriculum which the Melbourne Declaration of 2008 included, were replaced by another Ministerial statement of goals (the Alice Springs Education Declaration) which removed those references (Gough 2020).

Such fluidity in national support for climate change education notwithstanding, attention to environmental sustainability and climate change education by governments has been stimulated and supported by the growing interest of UN agencies on environmental sustainability and, more recently, on climate change in particular. The landmark 1992 United Nations Conference on Environment and Development, mentioned earlier, advanced a view of development inclusive of environmental sustainability that marked a turning point in the global environmental movement. *Agenda 21*, the action plan resulting from the conference included efforts in education to increase awareness of the concept of sustainable development. At the Conference, the United Nations Framework Convention on Climate Change was presented for signature, entering into force in 1994 once enough countries had ratified it. Article 6 of the convention underscores the importance of education and training to address climate change:

“EDUCATION, TRAINING AND PUBLIC AWARENESS

In carrying out their commitments under Article 4, paragraph 1 (i) the Parties shall:

- Promote and facilitate at the national and, as appropriate, subregional and regional levels, and in accordance with national laws and regulations, and within their respective capacities:
  (i) the development and implementation of educational and public awareness programmes on climate change and its effects;
  (ii) public access to information on climate change and its effects;
  (iii) public participation in addressing climate change and its effects and developing adequate responses; and
  (iv) training of scientific, technical and managerial personnel;
- Cooperate in and promote, at the international level, and, where appropriate, using existing bodies:
  (i) the development and exchange of educational and public awareness material on climate change and its effects; and
  (ii) the development and implementation of education and training programmes, including the strengthening of national institutions and the exchange or secondment of personnel to train experts in this field, in particular for developing countries.” (United Nations 1992, page 5).

A decade later, in 2002, the World Summit on Sustainable Development underscored the interdependence of various dimensions of development as part of the notion of sustainability. A full ten years later, in 2012, the UN Conference on Sustainable Development again underscored the interdependence of social, environmental and economic development and highlighted the lack of progress in integrating these three pillars (UNESCO 2016, p. 5).

In 2014, The Ministers and Heads of Delegation attending the twentieth session of the Conference of the Parties and the tenth session of the Conference of the Parties serving as the meeting of the Parties to the Kyoto Protocol, adopted a declaration that specifically underscored the necessity of determined educational initiatives to address climate change. The Lima declaration states:

“1. Stress that education, training, public awareness, public participation and public access to information play a fundamental role for all countries to achieve climate-resilient sustainable development and contribute to meeting the objective of the Convention;

2. Reaffirm our commitment to promote and facilitate at the national, subregional and regional levels the development and implementation of educational and public awareness programmes on climate change and its effects, of public access to information and of participation in decision-making on climate change;

3. Encourage all governments to include the issue of climate change in curricula and to include awareness-raising on climate change in the design and implementation of national development and climate strategies and policies;

4. Urge all Parties to give increased attention to the topic of education, awareness raising and public participation in all aspects of climate change negotiations;

5. Call on all Parties to re-emphasize the importance of education, training, public awareness, public participation and public access to information on climate change and its effects in the new global agreement to be concluded in Paris in 2015;

6. Reaffirm our commitment to cooperate and engage through bilateral and regional complementary initiatives that aim to raise awareness and enhance education on climate change and its effects.” (United Nations, Conference of the Parties 2014).

In September of 2015, at the annual general conference of the United Nations, the governments of the nations participating adopted a compact of development which embraced the goal of sustainable development, identifying seventeen goals and a series of specific targets. Sustainability was the driving concept of the entire framework, which highlighted the role of education, one of the sustainable development goals, in achieving all of the remaining goals, including Goal 13 ‘Take urgent action to combat climate change and its impacts’. Specifically, Goal 4 ‘Ensure inclusive and equitable quality education and promote lifelong learning opportunities for all’, includes a target (4.7) that explicitly focuses on education about sustainable lifestyles:

“By 2030, ensure that all learners acquire the knowledge and skills needed to promote sustainable development, including, among others, through education for sustainable development and sustainable lifestyles, human rights, gender equality, promotion of a culture of peace and non-violence, global citizenship and appreciation of cultural diversity and of culture’s contribution to sustainable development.” (UN 2020, Target 4.7.)

That same year, the United Nations Climate Change Conference in Paris approved the Climate Paris agreement, eventually adopted by delegates from 195 nations recognizing the importance of education for climate change education in Article 12.

At present UNESCO promotes Climate Change Education through its Education for Sustainable Development Program, established in 2010, through policy advocacy, facilitating exchange of good practice on climate change, supporting countries through capacity building, supporting the Associated Schools Network in climate action, and disseminating education resources. Salient among those resources is a guidebook to support whole-school approach to ESD and climate change, which involves mainstreaming sustainability into all activities of the school, including curriculum and teaching, advancing sustainability in facility management, school governance and cooperation with parents and community (Gibb 2016).

An analysis of the government reports submitted as part of the process of reporting under the UN Framework Convention on Climate Change, shows that climate change education is addressed by most countries, 95% of those reporting, largely through public awareness efforts that emphasize cognitive dimensions. There is, however, relatively limited emphasis on socio-emotional and behavioral dimensions. Among the 194 countries reporting to the UN Framework Convention on Climate Change, 95% of them report incorporating climate change education in one of their recent reports (UNESCO 2019a, p. 5). Half of those did incorporate climate change education in formal education settings. Considerably less attention was given to education of groups in government, industry, non-governmental organizations and the scientific community (UNESCO 2019a, p. 5). Those reports prioritize public awareness (47%) followed by education (17%) and training (15%). The reports on inclusion of climate change in formal education prioritized knowledge, followed by skills, and to a much lesser extent by socio-emotional skills. At the primary level, for instance, 67% of the reports were about knowledge, followed by 27% focused on skills and 7% on socio-emotional skills. At the secondary level, 63% focused on knowledge, 33% on skills and 4% on socio-emotional skills. At the tertiary level, 75% focused on knowledge, 0% on skills and 25% on socio-emotional skills (UNESCO 2019a, p. 7).

An in-depth analysis of policy documents in ten countries with an expressed commitment to Education for Sustainable Development and Global Citizenship Education undertaken by UNESCO, revealed that in all these countries there are abundant references to both of these concepts, and that they are expressed in terms of cognitive, socio-emotional and behavioral dimensions (UNESCO 2019b). In the documents examined in these countries—Costa Rica, Japan, Kenya, Lebanon, Mexico, Morocco, Portugal, Republic of Korea, Rwanda and Sweden—there were almost twice as many references to Global Citizenship Education (representing about 60% of the references) than to Education for Sustainable Development (representing about 30%) across national laws, strategic plans and policies, national curriculum frameworks, programmatic documents and subject specific curriculum. These references were present across various subjects in the curriculum, and the emphasis on cognitive dimensions, relative to socio-emotional and behavioral dimensions, increased in secondary education.

Growing attention to climate change education on government policy frameworks and curriculum has created space for the development of a practice of climate change education. However, this practice is very heterogenous and evidence on its effectiveness is contested. As a result, the field has not yet reached the point where there is a robust consensus on what the evidence indicates is good practice. This absence of a robust academic consensus contributes to the fragility of this field of educational practice, to disconnects between policy and practice, and to the vulnerability of the field to the shifts induced by partisan influence in education policy.

A recent review of environmental education research, for example, concludes that the focus of most empirical studies is on individual effects in energy conservation behavior among children and youth, with very limited attention to effects on collective action or on the kind of sociotechnical transformation necessary to move away from fossil fuel energy consumption to renewable based energy systems (Jorgenson et al. 2019). The reviewers argue that many of the existing Environmental Education approaches depend on dated approaches developed in the 1970s and 1980s which assumed that “environmental problems could be adequately addressed through resource conservation and incremental changes to technology and human behavior.” (Jorgenson et al. 2019, p. 160) The reviewers argue that this exclusive focus on individual behavior is inadequate to address climate change, “a systemic problem of such scale and complexity that addressing it requires systems level change that results from the interaction and coordination of actions and innovations across multiple levels of scale” (Jorgenson et al. 2019, p. 160). Of the 70 studies included in the review, which covered articles published between 2012 and 2018, less than a handful conceived of environmental/climate change education as influencing collective action.

By minimizing the role of collective action, environmental educators and researchers may be reinforcing a simplistic and narrow conception of the relationship between climate change, human action, and energy system change and distorting the fact that many of the most impactful climate actions are decisions about energy supply systems that are made by state and market sector actors under direct pressure from advocacy coalitions and other social collectives (Jorgenson et al. 2019, p. 166). Another review of the climate change education literature suggests the need for approaches which are socially transformative, focusing on empowering students to act. The review suggests that much climate change education is focused on helping students understand climate change, the science of climate change, but is insufficiently focused on helping them identify pathways to change climate change (Stevenson et al. 2017, p. 70).

Another recent review of climate change education studies, covering literature published between 1993 and 2014, concludes that many of these studies document very limited effects of the programs evaluated on students’ attitudes and behavior (Rousell and Cutter-Mackenzie-Knowles 2020). The authors of the review argue that largely missing from the literature are approaches to climate change education which are participatory, interdisciplinary, focused on affect and creative. This review of the literature on climate change education identified a tension between the more predominant knowledge-based approaches to science education and interdisciplinary, affect driven and experiential education. Among the former, focused on the development of scientific knowledge about climate science, some studies however found no relationship between scientific knowledge and pro-environmental behavior. In contrast, a number of the studies focusing on cooperative, interdisciplinary, placed-based, experiential programs showed that those impacted attitudes and behaviors towards climate change (Rousell and Cutter-Mackenzie-Knowles 2020, p. 196).

About half of the studies reviewed focused on fostering scientific knowledge-based instruction on climate change education, followed by an emphasis on curriculum and pedagogy. These two approaches are followed by behavior change approaches, emphasizing education approaches designed to influence individual behavior, and adaptation and mitigation approaches emphasizing minimizing the impact of climate change.

The approaches to climate change education which focus exclusively on the development of knowledge are based on limited models about the factors which influence engagement with climate action. Emotions have been found to be important correlates of active engagement with climate. A study of the relationship of hope concerning climate change with pro-environmental behavior among Swedish youth and young adults found that hope plus worry about climate change was positively related to pro-environmental behavior, whereas hope plus lack of worry was not (Ojala 2012). Given the important role of emotions such as hope and worry in pro-environmental action, Ojala has advocated that they should be cultivated to sustain actions that challenge existing norms and institutions that contribute to climate change. She explains that a transformative and transgressive climate change education that recognizes the worry and anxiety that disrupting and transgressing generates, can create hope through critical emotional awareness and through activities that develop visions of preferable futures (Ojala 2016, p 52).

While the field of climate change education lacks the scientific consensus which characterizes domains such as ‘literacy’ or ‘how people learn’ (reflected, for instance, in the consensus reports on these topics produced by the National Research Council in the United States) there are emerging efforts to synthesize ideas about what works in practice. For example, a recent review of literature on the practice of climate change education, conducted by the Alberta Council for Environmental Education (2017) identifies six key principles of excellent climate change education:

- Frame climate change education in ways that focus on solutions, rather than on problems, build a positive narrative around shared identity. Focus on energy, conservation and outdoors education. Rely on pedagogies which engage in deliberative discussions, promote exchanges with scientists, address misconceptions, and implement school and community projects
- Keep the audience in mind. Develop curriculum that is appropriate to the age of the child, support teachers.
- Design programs which are action oriented. Build the agency of students.
- Develop activities that extend beyond climate science, including imagining a positive desired future, focus on local content, teach students how to think, not what to think, do not scare students.
- Establish connections to the curriculum and identify competencies. Emphasize cross-curricular approaches, cultivate systems thinking, and help students understand the interdependencies between climate change mitigation, adaptation and resilience.
- Evaluate for program improvement.

In spite of this growing body of practice, and of the more limited body of research, documented levels of student knowledge and skills with respect to Climate Change or Environmentally Sustainable Education more generally appears to be inadequate to meet the urgency of the challenge, and as mentioned when discussing the reviews of this research, evidence of impact of climate change on attitudes or behavior is elusive.

The Program for International Student Assessment, administered by the OECD, shows that, on average, only one in five students in the OECD countries can consistently identify, explain and apply scientific concepts related to environmental topics (OECD 2012). Conversely, 16% of the students don’t have enough knowledge to answer questions containing scientific information related to basic environmental issues, and 20% of the students are just at that baseline level of scientific proficiency. These low levels of scientific knowledge and skills are in spite of the fact that all students in the OECD attend schools that report that they teach environmental science as part of the science curriculum. The latest administration of the PISA study revealed that less than 10% of all students tested could distinguish facts from opinions (OECD 2019, p. 3).

This evidence underscores the fact that the current challenge with climate change education is not just a challenge of including it in the curriculum, and most certainly not a challenge that is solved when governments include it in policy pronouncements, but it is also a challenge of developing the capacity of teachers to support deeper learning among their students. Deeper learning requires an integrated view of how knowledge relates to behavior. Such integrated views about the breath of competencies that undergird human functioning, extending them beyond knowledge, have evolved over the last few decades.

In 1991, UNESCO’s General Conference, proposed the creation of a Commission to develop a framework for education in the 21st century. The report produced by the commission proposed that education should be organized around four goals: learning to know; learning to do; learning to live together; and learning to be (Delors 1996). Building on UNESCO’s Delors Report, UNICEF developed a framework of life skills and citizenship to support the development of children in the Middle East that reflects an ambitious set of twelve core life skills aligned to the four pillars in UNESCO’s report. Learning to know, for instance, is reflected in Skills for Learning (creativity, critical thinking, problem-solving), learning to do in Skills for Employability (cooperation, negotiation, decision-making), learning to be in Skills for Personal Empowerment (self-management, resilience, communication) and learning to live together in Skills for Active Citizenship (respect for diversity, empathy, participation) (UNICEF 2017, p. 4).

The Organization for Economic Cooperation and Development also contributed to the global dialogue on broader goals for education through a Learning Framework outlining an expanded set of competencies that could contribute to individual and collective wellbeing (OECD 2020).

“The OECD Learning Compass 2030 defines core foundations as the fundamental conditions and core skills, knowledge, attitudes and values that are prerequisites for further learning across the entire curriculum. The core foundations provide a basis for developing student agency and transformative competencies. They are also the building blocks upon which context-specific competencies for 2030, such as financial literacy, global competency or media literacy, can be developed.” (OECD 2020, p. 2)

In 2019, UNESCO established a high-level commission to prepare a report on the Futures of Education. In a series of submissions to the commission by several holders of UNESCO’s chairs, many of them highlight the importance of educating for climate-change and advance ideas that emphasize going well beyond knowledge of the facts about climate change. Some of these authors argue that sustainability is an inherently cross-disciplinary topic, which requires an understanding of the systems which undergird climate change. This requires being able to integrate insights from economics, science and social science, but traditional curricular silos impede such understanding of systems (Jain 2020, p. 30). Such interdisciplinarity is necessary not just to cultivate understanding of systems among students, but also among scientists themselves. Given the importance of geology to understand sustainability, it is paradoxical that most geologists are not involved in sustainability science and their education seldom addresses sustainable development (Stewart 2020, pp. 39–40). In order to mobilize geology based knowledge to address human harm to the environment, requires collaboration with engineers, planners, biologists, zoologists, ecologists, agronomists, environmental scientists as well as social and behavioral sciences (Stewart 2020, p. 40). Educating for sustainability requires also cultivating “diversified and shareable imaginations of the territory and our living environments that contribute to the development of a viable future” (Poullaouec-Gonidec 2020, p. 33). The development of such imaginations of sustainable living environments requires dialogues that are anchored on the arts, sciences and humanities. Central to educating for sustainability is also to help students develop ethical frameworks which can value the environment and life in themselves, an education which helps students know the multiple humanistic traditions and that engages them with environmental ethics (Mantatov et al. 2020, p. 35). Educating to value the natural world, instead of material possessions, is also foundational to the development of ethics and imaginations that value nature and the environment.

The breadth of skills identified in these various frameworks underscores the importance of addressing more than knowledge of facts about climate change in order to prepare students to translate knowledge into action. The capacity to engage students in such deeper learning requires high level of skills from teachers, and a well developed curriculum, in order to not only engage their students in high cognitive activation tasks, but also to develop the socio-emotional competencies that undergird agency and the capacity to take responsibility for climate change and to collaborate with others productively in addressing it. The reviews of research on climate change education cited earlier show that such approaches to climate change education, while apparently effective, are rare.

In order to engage their students in deeper learning in this domain teachers need support to develop their own knowledge about climate change. A recent survey of a nationally representative sample of science teachers in the United States conducted by the National Center for Science Education (Plutzer et al. 2016) revealed that while three quarters of the science teachers address climate change in their classes, only half of them do so in ways that are aligned with the current scientific consensus. When asked to rate their own content knowledge with respect to climate change, ecology, modern genetics, weather forecasting and health and nutrition, 17% of the teachers report that they know less about this topic than most other high school teachers, and 31% report the same for weather forecasting models. Only 28% of the teachers report that their knowledge of climate change is very good or exceptional, compared to 45% who report this level of knowledge for ecology or 44% for genetics or 48% for health and nutrition (Plutzer et al. 2016, p. 19). When asked to select a series of possible topics to be covered to teach a unit on greenhouse gases and recent global warming, a topic which most teachers reported they taught and one on which the basic science on how these gases trap heat is a century old and noncontroversial, only some of the teachers selected as high priority topics which are essential to understand greenhouse gases. (Table 1.3)

**Table 1.3:** Priority given to potential topics in a teaching unit on the greenhouse effect. (Plutzer et al. 2016, p. 21)

“Imagine that you were asked to teach a 2–3 day unit on greenhouse gases and recent global warming. What priority would you give to including each of the following possible topics?”					
	A high priority	A medium priority	It is not necessary to cover this topic	This topic should not be covered	I do not have an opinion
Carbon dioxide trapping heat in the atmosphere	74%	22%	1%	0%	3%
Use of coal and oil by utility and electric companies	59	40	2	0	3
Emissions from industry	56	33	2	0	4
Destruction of forests	55	39	2	1	3
Depletion of ozone in the upper atmosphere *(foil)*	42	41	11	3	3
Incoming shortwave and outgoing longwave energy	24	39	15	1	20
Use of chemicals to destroy insect pests *(foil)*	23	42	23	5	?
People heating and cooling their homes	21	62	10	1	6
Use of aerosol spray cans *(foil)*	14	56	20	4	7
The impact of launching rockets into space *(foil)*	4	27	41	7	22

The same survey reveals that most teachers are unaware of the scientific consensus attributing global warming to human activities, with 61% of them demonstrating ignorance of such scientific consensus. Only 39% of the teachers in the study correctly recognize that over 80% of climate scientists think that global warming is caused mostly by human activities, and an additional 21% of the teachers admit that they don’t know the answer, with the remaining 40% providing an incorrect answer (Plutzer et al. 2016, p. 22). Teachers report that they have received very limited training on climate change, only 43% had any formal instruction on the subject at the college level, and only 10% completed a course on the subject (Plutzer et al. 2016, p. 23). Among those without education on climate change during initial preparation, only 18% received any professional development on the subject. Teachers recognize this topic as a high need for preparation, and 67% report that they would be interested in professional development opportunities on the subject (Plutzer et al. 2016, p. 24).

## The Limitations of Current Climate Change Education Efforts

In addition to the limitations stemming from too narrow a definition of the intended outcomes of climate change education (focused only on low levels of cognition with insufficient attention to higher order cognitive skills or to intra personal or inter personal skills) and of the approaches to achieve them (didactic and siloed into a single subject instead of problem and activity based and interdisciplinary), a second set of limitations explains the challenges of going to scale with effective climate change education programs.

As described in the previous section, many of the ongoing climate change education programs are based on an implicit or explicit top down model of change which assumes that if intergovernmental bodies and governments embrace the purpose of climate change education, this will transform instruction and learning. These efforts are misguided in two ways. First, they assume that climate change education is a technical challenge with a universal solution, that there is one content and modality of education which can be rolled out across all jurisdictions in the world. Technical problems require that the solution to the problems is known. But if climate change education were a technical challenge, and we knew how to solve it, we would not face the paradox described in the first section of this chapter given how much policy rhetoric has been devoted to it and how much attention governments profess to devote to it in their reports to UN agencies and in policy declarations. The simple question we must answer is, given that governments state their interest in climate change education, and given that there is an abundance of resources that offer guidance on how to do it, such as UNESCO’s guide on a whole school approach to climate change education, why isn’t it happening and why isn’t it achieving the necessary results? Why is it that “Despite efforts over the past 40 plus years, environmental sustainability is still on the margins of the curriculum in most countries.” (Gough 2016, p. 84).

International declarations, governmental agreements, and top down policy guidance on climate change education suffer from an inherent limitation. Effective programs of climate change education need to be designed to serve specific populations and to fit the particularities of institutional settings in unique jurisdictions. The need for contextually relevant approaches to climate change education stems from three facts. One, that climate change influences various locations and populations in unique ways. Two, that the systems that contribute to or mediate the impact of climate change are local, and changing them requires knowledge and skills that are fit for context and purpose. Third, that the approaches to climate change education need to fit the characteristics of the local education systems or schools.

I learned about these limitations of designing ‘generic’ curricula from my own work over a decade designing curriculum aligned with the United Nations Universal Declaration of Human Rights and Sustainable Development Goals. In 2009–2010, with a group of my graduate students, I developed a comprehensive curriculum, spanning from kindergarten to high school, aligned with the UN SDGs (we initially worked with the Millennium Development Goals, and later on substituted them with the Sustainable Development Goals as they were adopted at the UN General Assembly in 2015), with the Universal Declaration of Human Rights, and with the World Economic Forum Risk Assessment Framework. From the study of those goals, we developed a framework of competencies which a high school graduate should have in order to contribute to achieving such goals, we emphasized cognitive and socio-emotional competencies. Then, we used this framework to guide the development of 350 units to be taught in a special course, a ‘world course’, that would provide students explicit opportunities to integrate knowledge gained in various disciplines, as they worked on projects aligned with those competencies (Reimers et al. 2016). The book was well received and as a result many educators attempted to incorporate this curriculum in their schools. I discovered then that to do so they had to make significant adaptations to our original design, for instance, most could not devote the eight to ten hours a week, every week from kindergarten to high school, we had anticipated would be necessary to teach this curriculum. Most teachers ended just using this comprehensive curriculum as inspiration and as a resource, and adapted it in ways that fit with their existing goals and capacities. Even the first school that was genuinely motivated to adopt the ‘world course’, a newly established network of international schools (the Avenues School) ended up making successive adaptations to the original design.

Thinking that the challenge the ‘world course’ had encountered was one of complexity, in 2016, working with 36 of my graduate students, I developed a streamlined global education curriculum, from kindergarten to high school, following the same process of backward design from the UN Sustainable Development Goals (Reimers et al. 2017). This second attempt worked much better, in part because this time the book included not just a curriculum prototype, but a process of whole school change proposing how to introduce the new curriculum in the school and how to integrate it into the existing curriculum. One significant shift represented by this new curriculum is that I moved away from a dedicated ‘world course’ that required its unique place in the curriculum, dedicated teachers and eight to ten hours a week, every week from kindergarten to high school, replacing it with an approach that proposed to infuse global education widely across the curriculum. We offered a method to do this, and five lessons per grade which could be infused into existing subjects. This shift reduced some of the institutional demands to implement global education in the school, as it required no additional time and no additional faculty, even if it engaged considerably more people in the process, in effect increasing the demands. The approach differed also in that it made it possible to develop a whole school approach to sustainability education that did not see schools as blank slates, but that allowed schools to build more intentional sustainability education programming on already existing missions, plans, courses and activities. In an effort to convey that the process of whole school design of their own approach to sustainability education was more important than following any specific curriculum, I then developed, with a group of 34 of my graduate students, a variety of different curriculum prototypes, aligned with the UN Sustainable Development Goals (Reimers et al. 2018). My goal with this third curriculum resource was to convey that there were multiple pathways that schools could follow to empower their students as global citizens, that would be upstanders for sustainability.

Again I found that the most productive use of these resources was when teachers adapted it to their own needs, goals and capacities. Based on that work I then developed approaches to help teams of teachers, across schools, collaborate in developing their own adaptations of those curriculum resources, make them their own, and improve them through their practice.

From this work I discovered how important it was to attend to the details of implementing a new curriculum in a particular context. Using my book ‘Empowering Students to Improve the World in Sixty Lessons’ as a starting point, I have worked with networks of teachers in developing global education curriculum, such as the Rete Dialogue, a network of teachers in Italy committed to democratic education, in translating and adapting this book to the Italian context. Over a year, this network of teachers translated the original book, taught these lessons, and then modified them, as part of a learning community in which they collaborated in this process across various regions in the country. The result of this process was a revised curriculum, reflecting the learning these teachers had drawn from their practice in experimenting with the original lessons (Reimers et al. 2018).

Similarly, working with a group of fifty teacher leaders supported by the National Education Association Foundation in the United States, we developed a curriculum, inspired by ‘Empowering Students to Improve the World in Sixty Lessons’ in which teams of teachers from all US states collaboratively designed grade specific lessons aligned with the UN Sustainable Development Goals, taught them in their respective schools, and then improved based on their various experiences teaching them. This year long collaborative project, relying on the use of communication technology, led to two publications developed with two different group of teachers which they then used to further advance global education in their schools (Reimers et al. 2018, 2019).

These experiments designing and supporting the adoption of a sustainability curriculum in a variety of schools in different countries taught me that much more than curriculum was necessary to support a change in the culture of education.

Developing an approach to climate change that is fit for context is analogous to designing a new toilet that is fit for particular contexts, to use the example described earlier, rather than to transplant a universal design of a toilet from one setting to another. While there may be general principles of climate change curriculum that can be usefully taught across the world, the contextual nature of the impact of climate change and of mitigation approaches limits the value of universal curricula and approaches.

The second way in which many of the existing efforts to mandate climate change education from on high in national governments are misguided is that this top down approach to climate change education is equivalent to thinking that if we only wished for climate change education to happen, these wishes would trickle down to every school around the world. Trickle down climate change education does not work, even if it is enhanced with a few demonstration schools and with some one size fits all instructional materials.

As with other failed efforts to transform educational practice, the chief shortcomings of ongoing climate change education efforts are in underestimating the capacity and institutional requirements at the point of delivery: the classroom and the school, or the instructional setting of the nonformal education program. For example, UNESCO’s guide on using whole school approaches to climate change does not include a single word on how to support teachers to develop the capacities to teach climate change. Similarly, a recent review of 221 studies on climate change finds that the topic of teacher professional development is conspicuously absent, and the few studies that addressed it focused on pre-service teacher preparation rather than on professional support for practicing teachers (Rousell and Cutter-Mackenzie-Knowles 2020, p. 200). A similar conclusion is reached by the authors of a recent study of the place of environmental sustainability in teacher education programs in Canada, who found that over the last four decades, Canadian faculties of education have faced many challenges incorporating environmental sustainability education, and as a result the topic remains a very peripheral concern in pre-service as well as in-service teacher education programs (Karrow et al. 2020, p. 2).

The same conclusion that professional development is an afterthought, or entirely missing, from efforts to advance environmental education is reached by the author of a study of the history of environmental education in Australia and England who found that, even when the curriculum included the goal to make sustainability a priority “the actual content of the four core areas of the Australian Curriculum (English, History, Mathematics, and Science) does not enact the statement’s intent, nor is there guidance for teachers in implementing the Organising Ideas for Sustainability” (Gough 2016, p. 89). This study concludes that the ambitious framing of environmental education as a cross-curricular priority in practice was reduced to eight areas of content in the subject of science, and found no specific strategies to support teacher collaboration across subjects. (Gough 2016, p. 91).

Notwithstanding the fact that international agencies and governments seem to assume teacher capacity to teach climate change, the development of effective climate change curriculum appears to exceed the pedagogical capacities of most teachers, schools and education systems, and hence a new climate change curriculum cannot be ruled by decree, but must instead be supported with opportunities for teachers to develop new knowledge and capacities. This is simply a corollary of what is known about how to build instructional capacity for deeper learning and twenty-first century education (Reimers and Chung 2018; Reimers 2020a).

As is true of other ambitious efforts to change the curriculum, such as educating for global citizenship, in order to transform educational practice, the design and implementation of climate change education programs needs to be approached multidimensionally, attending to the cultural, psychological, professional, institutional and political dimensions of the change process (Reimers 2020b, c).

Transforming school culture at scale, for large numbers of schools in education systems, will require more than effective curriculum and professional development. In a recent study of approaches to global citizenship education I argue that past approaches to global education have failed because they have adopted too narrow a view of the process of change, focusing on curriculum, or on teacher professional development, but failing to simultaneously address the change process through the five dimensions which characterize it: cultural, psychological, professional, institutional, and political. These five perspectives integrate what is known about how students learn and how schools change (Reimers 2020b).

“The cultural perspective, for example, defines the broader set of societal hopes for schools, norms and values which define what are accepted educational goals and practices. The psychological perspective illustrates the theories of learning which undergird the learning and teaching process. The professional perspective focuses on how expertise is inserted in professional roles to advance teaching and learning. The institutional perspective attends to the various structures, processes and resources that provide resiliency to the system of education, governing the interactions among the actors that form the system and providing stability and meaning to teaching and learning. The political perspective illustrates how the interests of various groups are negotiated and conflicts resolved, resulting in a particular culture of education.” (Reimers 2020b, page 8).

“Together, these five perspectives illuminate the complete process of change as the partial elements highlighted by each perspective offers a perspective that complements what other perspectives enlighten and, together, these various elements brought to light by each perspective interacts with the elements highlighted by other perspectives. Paraphrasing Goethe who said that the person who speaks with only one language sees the world with one eye, thinking about educational change through a singular frame is seeing change with one eye. A multidimensional model thus helps capture the gestalt of the process of educational change and provides depth, perspective, a fuller and more complete understanding.” (Reimers 2020b, page 22).

A cultural perspective requires that we understand the relationship between a program of climate change education and the expectations and understanding of the local community, and that we engage them in broadening the zone of acceptance of what can be taught so that the curriculum can make room for climate change education. Many students and parents are already aware of the urgency of addressing climate change and, in many settings, they are likely to be allies, supportive of climate change education efforts. A recent survey of a representative sample of parents and teachers in the United States, found that 74% of the teachers, and 68% of the parents, believe that schools should teach about climate change and its impacts in the environment, economy and society. An additional 12% of the teachers and 16% of the parents believe that climate change should be taught, but not its impact. In spite of such support for teaching climate change, only 42% of the teachers and 45% of the parents in the same survey talk to children about climate change (Kamenetz 2019).

A psychological perspective draws on the advances of the learning sciences to design learning experiences that effectively help students gain the deep understanding necessary to inform and motivate behavioral changes. A professional perspective focuses on how to advance climate change education in a way that reflects the best expert knowledge on climate change, and in a way that strengthens the professional capacities of teachers and other educators.

An institutional perspective focuses on the systemic nature of the process of change, requiring alignment between curriculum, assessment, instructional resources, teacher support, and leadership.

A political perspective focuses on identifying the various stakeholder groups affected by climate change education and their position with respect to a climate change education program, and with creating opportunities to mobilize as much support as possible and address the interests opposing it (Reimers 2020b).

When it comes to addressing themes which may challenge prevailing views, or dominant political structures, teachers and schools must tread lightly, as there are boundaries to the educational ideas and goals that communities will find acceptable. This shouldn’t mean that teachers falsify the available scientific consensus with respect to climate change or the fact that most scientists agree it is caused by human activity if powerful interest in their community are opposed to teaching such scientific consensus, but it underscores the necessity of developing synergies between school-based efforts to address climate change and non-formal education programs. Communities need to be educated as well in order to expand the zone of acceptance of what schools are authorized to do.

The failure to address educational change through a cultural and political perspective can lead to implementation processes which transforms curriculum and policy in dramatically undesirable ways. For instance, in the United States the politization of discussions of climate change leads teachers to teach content which deviates from the scientific consensus. A recent study of the National Center for Science Education of how teachers teach climate change in the US found that whereas three quarters of the science teachers did address climate change in the curriculum, only 54% did so in ways which were aligned with the scientific consensus. In contrast, 10% of the teachers taught incorrect knowledge, such as the ideas that recent increases in temperature are due to natural causes and teach that it is not the case that the scientific consensus establishes that global warming is primarily being caused by human release of greenhouse gases from fossil fuels. An additional 31% of the teachers sent mixed messages in their teaching, correctly teaching that the scientific consensus is that that recent global warming is primarily being caused by human release of greenhouse gases from fossil fuels, but incorrectly teaching that many scientists believe that recent increases in temperature are likely due to natural causes (Plutzer et al. 2016, p. 16).

The challenge of climate change education thus becomes a challenge of augmenting the capacity of education systems and schools to develop and teach high quality curriculum, evaluate it, and develop and implement high quality programs of teacher education that support teachers in adopting more effective pedagogies of climate change education. This challenge of capacity in classrooms and schools must be addressed in order to scale a new set of instructional practices and a new culture of education that support climate change education. The culture of schools does not change because governments subscribe international conventions, or because they include climate change education in their policy pronouncements or in the curriculum or even because they distribute lesson plans to schools. Transforming the culture of education to advance climate change education requires that we think about the enterprise through a multidimensional framework that attends to the cultural, psychological, professional, institutional and political aspects of the enterprise (Reimers 2020b). Teachers and school leaders need knowledge and skills to effectively deploy programs of climate change education addressing these five dimensions of the process, and this is currently absent in many schools.

## The Need for New Strategies for Climate Change Education

The paradox presented by the fact that in spite of the recognition that education can address climate change our efforts have been insufficient to address the most serious risks posed by human-made environmental degradation, should not lead us to abandon our efforts, or to conclude that education is irrelevant to the challenge of climate change. Rather, it should cause us to rethink our approach to this adaptive challenge. This strategic rethinking of education for climate change needs translate into situated responses focusing on who should be educated, what the focus of such education needs to be, and how such education should be delivered. Because the ways in which climate change impacts people vary in different jurisdictions, and because the characteristics of educational institutions available to educate vary in their reach and capacity, education strategies need to be developed which are specific to specific education contexts. International organizations are better at forging agreements than they are at designing and supporting the implementation of context specific climate change education strategies in particular localities. As we have seen such efforts in policy rhetoric serve a useful, but insufficient, role. They open up space for climate change education, but they do not produce effective climate change education. Context-agnostic guidance is of limited use to effectively support the implementation of changes that transform the capacity of teachers, schools and non-formal education institutions so they can help all gain the knowledge, skills and dispositions to adapt to, mitigate and revert climate change.

Many different populations across a variety of jurisdictions must be educated to address climate change. They include children and youth, certainly, as well as adults in their various occupations and roles so they can from within those roles adopt practices that are more sustainable. In their roles as consumers, farmers, factory workers, commuters, community members, business owners, CEOs or political leaders or activists, each person needs to not only understand climate change, but have the skills to translate that knowledge into implications for their own behavior, and the capacity to make a commitment to acting in more sustainable ways, including not just individual consumption but collaboration with others and collective action to transform systems. Climate change education needs to involve school-based and university-based programs, as well as non-formal and informal education modalities. It needs to educate old and young, the employed and the un-employed, leaders and followers, business owners and workers.

To be sure, there is a need to educate people on the scientific consensus with respect to climate change, as summarized for instance by the National Aeronautics and Space Administration Agency (NASA 2020). But knowledge of facts based on scientific consensus alone is likely to be insufficient as we have seen. People need the kind of deeper knowledge that can help them think and act in more environmentally sustainable ways. Education for climate change must therefore cultivate the broad range of skills which contemporary frameworks posit are necessary to participate in a rapidly changing world.

In order to be more effective at supporting such deeper learning, climate change education requires adequate supports to build the capacities of teachers and schools to advance pedagogies that foster high cognitive activation and that develop life skills to translate such knowledge into changed patterns of behavior. These efforts in schools and universities need to be enhanced with similarly effective non-formal programs which reach most adults. In other words, we need to considerably augment the intensity and efficacy of climate change education efforts at the point of closer proximity with learners around the world, at multiple points of delivery. We will not curb climate change with a few pilot projects, or with small networks of schools committed to the enterprise, or with episodic media campaigns or with pronouncements from international organizations or governments, or with curriculum intentions.

We also need a rigorous assessment of the underlying logic of ongoing efforts, of the underlying program theories of how certain actions are expected to achieve the results we seek, and a shift in mindset that makes visible how we weigh various criteria involved in making individual and collective choices and that give greater weight to sustainability in human behavior.

A context specific strategy needs to address these questions:

1. What are the specific impacts of climate change in this jurisdiction? How do they impact various human populations? Which of these populations needs to be educated on climate change?
2. What knowledge, dispositions and behaviors could mitigate the impact of climate change and are there ways in which changes in the behaviors of populations in this jurisdiction could slow down climate change?
3. What are the means of delivery to reach each of the specific populations in this jurisdiction?
4. What curriculum can best educate each population?
5. What institutions can support the development of the institutional capacity necessary to deliver such curriculum effectively?
6. What institutional collaborations can support the implementation of this strategy?

Developing and implementing such a comprehensive strategy is an adaptive challenge that will require a multi-stakeholder coalition that can produce collective leadership as no single institution has the authority, the power or the capacity to alone address the multiple efforts required. The design of such a strategy requires addressing efforts to advance climate change education attending to the cultural, psychological, professional, institutional and political dimensions of the enterprise (Reimers 2020b). Universities are singularly positioned to work with local governments, and with schools, to augment the capacity necessary to develop and deliver climate change education curriculum.

## The Need for Systemic, Multilevel and Multidimensional Perspectives In Climate Change Education

If climate change education is to support deeper learning among students, it cannot be a simple add on to the curriculum, a new silo where students access new content in some subjects. It certainly cannot be limited to providing students with knowledge of the facts about climate change or even with an understanding of how the systems that impact climate function. The reviews of policy and practice discussed earlier suggest that the earlier efforts of Education for Sustainable Development and Climate Change Education addressed the task primarily as developing new cognitive skills, as providing students with more knowledge. As understanding of curriculum has evolved to embrace a whole-child approach, and to focus on the breadth of skills essential to participate in rapidly changing contexts, Climate Change Education efforts are also embracing a multidimensional view focusing on cognition, socio-emotional and behavioral dimensions.

Climate change education curriculum needs to be aligned with such contemporary frameworks of twenty-first century skills and deeper learning in order to contribute to adaptation, mitigation and reversal of climate change, and teachers need to be supported to develop their skills in engaging their students in deeper learning about climate change.

Effectively creating the opportunities for the development of that breadth of skills with respect to climate change will require the type of whole school approach to change advocated by UNESCO (Gibb 2016). Supporting schools in enacting those changes will require strategies that are responsive to specific contexts, and that include effective professional development for teachers and school leaders. A recent study of programs which successfully prepare teachers to educate the whole child in various countries around the world concludes that they all indeed adopt a whole school approach to educational improvement (Reimers and Chung 2018). In addition, these programs have the following characteristics:

- They reflect a conception of adult learning that sees it as socially situated and responding to current needs of teachers for learning.
- They involve sustained and extensive opportunities for teachers to build capacities, often extending an entire school year, or spanning across multiple school years
- The modalities of professional development are varied. They include independent study of new material, discussion with peers and others, individual or group coaching, demonstrations of new practices, independent research projects and opportunities for reflection.
- The curriculum of the programs examined covers a blend of capacities, from a broad focus on helping students develop particular capacities to a highly granular identification of particular pedagogies and instructional practices that can help students gain those skills.
- The curriculum of these various programs reflects a view of learning which includes cognitive skills, in interaction with dispositions and socio-emotional skills.
- Professional development includes exposure to visible routines, protocols and instructional practices, where teachers see in practice new forms of instruction or assessment.
- These programs rely on a mix of opportunities for learning situated in the context of the schools where teachers work.
- To support the intensive and sustained activities of professional development that these various programs advance, the organizations in charge build a range of partnerships with institutions outside of schools that contribute various types of resources.
- These programs see teacher practice as situated in specific organizations and social contexts, and in general adopt a whole-school approach, rather than helping individual teachers increase their capacity.
- These programs all develop capacities among teachers to advance pedagogies with the goal of developing competencies that are not formally assessed in the school or school system. The organizations that support these various programs all model a learning orientation.

## A Role for Universities Developing and Implementing Contextually Appropriate Strategies for Climate Change Education

The specific impacts of climate change in particular communities and geographies differ, as do the ways in which particular communities contribute to climate change. As a result, the way in which people need to adapt to climate change, mitigate the impacts of climate change on their lives or diminish their impact on climate change need to be fit to context. For this reason, it is necessary that climate change education is situated in particular places, in particular geographies and economies. Whether the goal is to educate students for individual or collective actions, those have to be relevant to specific contexts. Generic lists of suggestions or teaching guides are of limited value to address these contextual specificities.

In addition, the institutional characteristics of education systems, schools and teachers differ in terms of their strengths and shortcomings, in some settings teachers are better educated than in others, some schools are institutionally weaker than others. Schools and systems also have different strengths, and any change process needs to build on those particular strengths. This wide variation in the context of schools limits the value of ‘one size fits all’ approaches to curriculum or teacher preparation for climate change education. While there is knowledge that is of universal value, such as knowledge about the science of climate change, translating that knowledge into a process that can develop particular competencies that matter to mitigate or adapt to climate change requires localization as does developing specific curriculum for particular teachers or particular students.

The transformation of institutional culture in order to effectively educate for climate change is too ambitious a task to be undertaken by schools or non-formal education organizations in isolation. They are too small to have the resources necessary to develop high quality curriculum and to, on their own, enhance the capacity of their teachers or facilitators to do this well. Such an effort requires a level of scientific expertise and innovation that calls for a larger scale and quality of resources than are typically available inside single schools. For example, there are many online resources that can be usefully deployed to teach about climate change, but curating these resources is one task that can take considerable time, for this reason assistance from university students in making thoughtful selections among the vast resources available in universities, and in the world wide web, to support teachers identifying suitable curriculum resources would be helpful.

It may also be unreasonable to ask of teachers that they single handedly take on the local or national politics which muddle the conversations about climate change with ideas that are extraneous to the scientific consensus. Engaging teachers in networks with other educators, and with colleagues in universities including scientists, can leverage the support of a professional community in developing teacher capacity to engage professionally with the topic.

If more scale is necessary to innovating in Climate Change Education than the scale available to single schools, why not leave the job to Ministries and Departments of Education? Because most departments of education have been designed to administer large systems, to manage resources efficiently and to ensure accountability, not to innovate. This is the reason most innovations in curriculum involve participation of other institutions and actors: disciplinary specialists, universities, professional associations.

Given the challenge of building teacher capacity, and given that Ministries of Education have proven that they are not well equipped to promote the necessary innovation to sustain high quality climate education at scale, where can the institutional resources to address this challenge be sought? A way to address this shortcoming in capacity is to repurpose an existing institutional resource in service of climate change education. That institutional resource are universities. Universities are ideally suited to take on this role of strengthening the capacity of schools and school systems to advance climate change education, translating the science of climate change, and the knowledge about what works for deeper learning, into specific programs that are tailored to concrete students, teachers and schools. Universities can do this in partnership with schools or non-formal education institutions that are within their immediate vicinity, or with schools that are situated more remotely if universities have ties of sufficient depth to those localities.

Clearly educating the public and supporting more sustainable choices requires also engagement of media, employers and governments. Universities, however, are unique in that they educate those professionals who work in those industries and others, potentially having a significant multiplier effect over time. Universities are also uniquely suited to educate about climate change because they contain, within the various departments of physics, chemistry, biology, the disciplinary expertise to develop high quality curriculum that reflects the current scientific consensus. They also have the scientific expertise to think about development comprehensively, and therefore to help students understand climate change in the broader context of poverty reduction and sustainability. Just as important, they also house, within their education departments, the expertise to translate the scientific knowledge base into effective k-12 curriculum and programs of teacher professional development. Drawing on collaboration across various disciplines, they can develop instructional resources that support the sophisticated thinking and action necessary for adaptation, mitigation and reversal of climate change. For instance, departments of public policy and social sciences can develop simulations that engage students in negotiating different views with respect to various policy proposals such as caps on emissions or carbon tax. As some of the competencies which students need to develop to think through options in response to climate change are ethical, there is also an important role for the humanities in cultivating the moral imagination of learners as they develop responses to climate change. Universities are the most capacious institutions for this kind of interdisciplinary collaboration in developing a rich curriculum and transformative pedagogies that support deeper learning about climate change.

Furthermore, the ubiquity of institutions of higher education around the world, makes it possible to advance efforts of curriculum development and capacity building which are responsive to the particular needs and opportunities of local contexts. An additional reason why universities are a strategic resource to support schools in addressing climate change is because they can integrate teachers into larger networks which provide them the intellectual resources and the support to understand that they are not alone in teaching about this topic. This support may be especially valuable in contexts where the politization of the subject of climate change challenges teachers.

An additional reason for universities to engage in this work, is that engaging in designing approaches to climate change education will teach university students how to solve problems and design innovations around climate change. Engaging students in problem-based learning, in specific contexts, helping them unravel the systems that undergird current climate predicaments in particular locales will help them learn to change them. In engaging them with local actors, this form of climate change education will also help students develop the skills for collaboration that will serve them well to continue to advance the necessary systemic change to curb climate change. These are the opportunities to gain the skills to understand and transform systems which a recent review of research on climate change and energy education is largely absent from most of the efforts they studied (Jorgenson et al. 2019).

Universities are already concerned with climate change and advancing a range of actions to address it, although more intentional and effective instruction is necessary in order for all university students to gain the necessary skills to understand, adapt to and mitigate climate change (Leal Filho and Hemstock 2019). A recent survey administered to university leaders and faculty working on sustainability in 51 countries reveals a gap between the priority universities attach to climate change and the generalization of opportunities for all students to learn about climate change (Leal Filho et al. 2019). Whereas 59% of the respondents agreed or strongly agreed with the statement that their university attaches strong priority to matters related to climate change, only 41% of them agreed or strongly agreed with the statement that there are opportunities to learn about climate change in the courses chosen by students, and 58% indicated that there are courses related to education for sustainable development available in the university curriculum. These responses indicate that climate change education is an option, but not a requirement, in many institutions of higher education (Leal Filho and Hemstock 2019, p. 7). Unsurprisingly, given that the respondents included faculty and senior staff working on sustainability, these responses about existing opportunities to learn about climate change contrast with the much higher number, 96% of respondents to the same survey, who agree or strongly agree with the statement that ‘a university should encourage its students to search for solutions with regards to problems caused by climate change’ (Leal Filho and Hemstock 2019, p. 7).

There are examples of the power of such collaborations between universities and schools to support more effective curriculum and instruction. In the United States, the new science standards were designed as a result of a collaboration between the National Research Council, the National Science Foundation, the American Association for the Advancement of Science and the National Science Teacher Association. In Chile, one of the most successful programs of inquiry-based science education in public schools was designed by science professors at the Universidad de Chile, inspired by a similar program developed by the Smithsonian Institution in the United States. In France, one of the leading organizations to support the improvement in the quality of science and technology teaching at the elementary levels, the Foundation ‘La main a la pate’ was established by the Academy of Sciences, and the Ecole Normale Superieure of Paris and of Lyon. The school of earth, energy and environmental sciences at Stanford University has designed a very high quality climate change education curriculum, which reflects the level of scientific rigor that results when scientists studying the topic translate what they know for lay audiences (Stanford University 2020). The National Aeronautics and Space Administration Agency has synthesized most of the scientific consensus, generated in universities, with respect to climate change (NASA 2020). Simply put, universities have unparalleled intellectual resources to develop climate change education curriculum and programs of teacher professional development that can support the necessary development of capacity that the enterprise demands. Furthermore, they have the know-how and the practice to experiment, evaluate, conceptualize and theorize which are essential to helping develop the field of climate change education into one where what is currently an largely undertheorized practice develops into a professional field of practice, into a field guided by expert knowledge and supported by the powerful tools of logic and science.

## Development of the Approaches to Climate Change Education in This Book

The purpose of this book is to inspire university faculty, students and leaders to integrate climate change education into existing courses, in ways which provide students opportunities to design approaches to climate change education which could be implemented in schools and non-formal education institutions at the precollegiate level. Doing this would serve two purposes. One to educate university students themselves so they learn how to design solutions to existing problems, rather than merely contemplate them. This is of intrinsic value to students in higher education, regardless of the specific problems they learn to solve. It is an approach to support deeper learning and the development of twenty first century skills, and to help students develop hope and self-efficacy in tackling challenging problems. The fact that climate change is one of the most critical challenges of our times, makes it especially important to cultivate students’ capacity to be change agents, rather than bystanders.

The other purpose this approach would advance is to augment the capacity of primary and secondary schools to educate about climate change, by relying on their partnership with universities. If only ten percent of the more than twenty thousand institutions of higher education around the world engaged only one professor each year in such an effort, each engaging five teams of students in the design of approaches to climate change education, this would produce each year 10,000 context specific strategies which could be offered to local coalitions for validation, adaptation, implementation and evaluation. The equivalent of 2,000 books like this one a year. This likely exceeds all the resources created by all international development institutions to support climate change education since the United Nations Sustainable Development Goals were adopted in 2015.

Over time, sustaining such efforts would build considerable expert knowledge about how best to educate communities to adapt to, mitigate and redress climate change. It is hard to imagine a more capacious engine than universities to design, research and support the development of capacity that this field needs with urgency in order to close the institutional capacity gap at the root of the climate education paradox. A production of 2,000 resource books a year systematizing climate change education would certainly contribute to theorizing the practice of climate change education and building this field of research and practice.

The next five chapters in this book exemplify context-specific education programs on climate change. They were developed in the context of a graduate course on education policy analysis I teach at the Harvard Graduate School of Education. The course covers the subject of policy analysis and design, as well as substantive themes focused on deeper learning, system level reform, curriculum, and teacher preparation. The course offers students the opportunities to work on several real-life projects, including consulting for governments and developing an approach to address climate change for a specific setting. To approach this task, students had to identify a real institutional partner for their work, an institution with an interest on the topic of climate change in a particular geographic setting. The students then studied the specifics of the particular challenge of climate change in that setting, the unique strengths and needs of the institution, and considered various alternatives to addressing the challenge (Bardach and Patashnik 2016). In effect, they addressed the key questions which I argue are essential to develop a context specific strategy of climate change education:

1. What are the specific impacts of climate change in this jurisdiction? How do they impact various human populations? How do human activities contribute to climate change?
2. What knowledge, dispositions and behaviors could mitigate the impact of climate change and are there ways in which changes in the behaviors of populations in this jurisdiction could slow down climate change? What kind of collective action could influence systems that contribute to climate change?
3. What are the means of delivery to reach each of the specific populations in this jurisdiction who needs to be educated on climate change?
4. What curriculum can help educate each population?
5. What role can the institution we are collaborating with play in advancing climate change education in that jurisdiction?

Students remained in communication with this institutional partner as they examined the root causes of the problem and designed and evaluated several approaches to addressing it, in an iterative process that involved loops of analysis-design-feedback over an entire semester. They then wrote a paper which described and analyzed the curriculum they had produced, conceptualizing their practice and integrating this work with the literature on climate change they had studied to support their design. Once their paper was completed, all authors of the chapters included in this book reviewed the entirety of the approaches developed to ensure greater coherence and alignment across the entire book, using this introductory chapter to guide those revisions. The papers were then presented at a global education conference at which leaders in the field of international development, including education specialists from UNICEF, the President of an international development organization and a former secretary of education of Mexico provided feedback to drafts of these chapters. They also received feedback to the programs they had developed from their partners and revised their chapters based on that first round of feedback. Once the full manuscript was finished, we received additional feedback from the editors of this series at Springer, Annette and Noel Gough, and from two anonymous reviewers, and made additional revisions to the chapters based on it.

The authors of these papers are educators with professional experience as teachers, child advocates, trainers, leadership mentors, curriculum developers and writers in Australia, Cambodia, China, Guatemala, Honduras, Japan, Nicaragua, Colombia, Pakistan, South Korea, Thailand, Uganda, United Kingdom, and the United States.

The programs they designed illustrate context specific climate change education programs, focusing on schools, non-formal settings and educator preparation institutions. The chapter are written with the aim of offering examples of general value beyond the specific contexts for which they were designed, but the essence of each chapter remains that, to be useful, climate change education needs to be firmly grounded in the specifics of a context and to be responsive to that context. We hope this level of detail, and the conceptualizations offered to justify the curricular and design choices which were made, will make these materials more useful to those who seek to adapt them to their particular contexts, or develop their own programs drawing inspiration from these examples.

Chapters 10.1007/978-3-030-57927-2_2 and 10.1007/978-3-030-57927-2_3 focus on climate change education in schools, each representing an approach to climate change education: a specific curriculum and a whole school approach to change. In contrast, Chaps. 10.1007/978-3-030-57927-2_4 and 10.1007/978-3-030-57927-2_5 focus on non-formal settings, Chap. 10.1007/978-3-030-57927-2_4 develops a specific skills-building radio education program whereas Chap. 10.1007/978-3-030-57927-2_5 develops a climate-change education skills program integrated into a larger literacy and life skills program in Pakistan. Chap. 10.1007/978-3-030-57927-2_6 focuses on preparation of graduate students in education, preparing for a variety of roles in ‘whole of education system’ reform.

In Chap. 10.1007/978-3-030-57927-2_2 ‘Learn to Lead: Developing Curricula that Foster Climate Change Leaders’ David Rhodes and Margaret Wang focus on the question of how to develop leadership capacities that help students in Israel and Palestine address climate change. Their approach to curriculum development engages an institution of civil society that brings together science students from Israel and Palestine to work on climate related topics, in the process of supporting schools. The curriculum emphasizes deeper learning and the development of transferable skills, and not just the transmission of factual knowledge.

In Chap. 10.1007/978-3-030-57927-2_3 ‘Creating a Culture of Shared Responsibility for Climate Action in Guatemala through Education’ Lina Lopez Lalinde and Carrie Maierhofer develop a whole school approach to climate change education in Guatemala. They explain why following UNESCO’s school-based approach of whole-school transformation is sensible, in a context in which climate change education is already included in the curriculum but not really implemented because of inadequate capacity at the school level, and examine the requirements of such an approach to building teacher capacity, proposing ways to address them relying on partnerships between government and organizations of civil society.

In Chap. 10.1007/978-3-030-57927-2_4 ‘Rezistans Kimatik. Building Climate change resilience in Haiti through educational radio programming’ Ashley Bazin and Christelle Saintis examine the challenge of responding to specific climate related challenges to vulnerable populations in Haiti, and opt for a non-formal radio-based approach to educate youth and adults.

In Chap. 10.1007/978-3-030-57927-2_5 ‘Adaptation, Migration, Advocacy. A Climate Change Curriculum for Out-of-School Children in Badin, Sindh’ Natasha Japanwala develops a non-formal education program to educate youth in a region of Sindh, Pakistan, where traditional agricultural livelihood is challenged by salinization of land for adaptation to those changes and mitigation of their impact in their lives.

Finally, in Chap. 10.1007/978-3-030-57927-2_6 ‘Students as Partners. Implementation of a Climate Change Education within the Harvard Graduate School of Education’ Annie Nam and Sueyoon Lee discuss the potential of student led curriculum development on climate change in schools of education, and develop a prototype of such a program for students at the Harvard Graduate School of Education.

In the concluding chapter I extract lessons and implications from these projects for future university based instruction of the sort I adopted in this course.

Our hope is that these five curricula will add to the ongoing knowledge base about climate change education, offering specific examples of how to advance this important area of education in ways which are responsive to context. We also hope that the entire book will illustrate the potential of engaging universities in designing climate change education curriculum, as a way to augment the capacity of schools, to develop necessary context specific approaches, and to educate university students to invent solutions to this defining issue of our times.
